# Examining the significance of arginine vasopressin release to elucidate the often multifactorial etiology of hypotonic hyponatremia: A novel criterion

**DOI:** 10.14814/phy2.15967

**Published:** 2024-04-07

**Authors:** Philip J. G. M. Voets

**Affiliations:** ^1^ Department of Internal Medicine University Medical Centre Utrecht Utrecht The Netherlands

**Keywords:** analysis, arginine vasopressin, criterion, hypotonic hyponatremia, test

## Abstract

Clinical hyponatremia guidelines, protocols and flowcharts are a convenient means for clinicians to quickly establish an etiological diagnosis for hyponatremia, and facilitate its often complex analysis. Unfortunately, they often erroneously attribute multifactorial hyponatremia to a single cause, which is potentially dangerous. In this manuscript, a novel criterion is proposed to quickly determine the physiological relevance of non‐osmotic arginine vasopressin (AVP) release, and to add nuance to hyponatremia analysis. While analyzing hypotonic hyponatremia, it is imperative to not only verify whether or not a certain degree of inappropriate AVP release is present, but also to ascertain whether it—in itself—could sufficiently explain the observed hyponatremia, as these two are not always synonymous. Using well‐known concepts from renal physiology to combine the electrolyte‐free water balance and solute‐free water balance, a novel physiological criterion is derived mathematically to easily distinguish three common hyponatremia scenarios, and to further elucidate the underlying etiology. The derived criterion can hopefully facilitate the clinician's and physiologist's interpretation of plasma and urine parameters in a patient presenting with hyponatremia, and warn against the important clinical pitfall of attributing hyponatremia too readily to a single cause.

## 1 INTRODUCTION

The plasma sodium concentration is a reflection of the total body water homeostasis, and its usual reference range is 135–145 mmol/L (Hall & Guyton, 2016; Mount, 2018; Verbalis, 2003). Hypotonic hyponatremia (hereafter: hyponatremia), defined as a plasma sodium concentration lower than 135 mmol/L with a low plasma osmolality, is the most frequently encountered electrolyte disturbance in hospitalized patients, and results from a net gain of electrolyte‐free water (EFW). Its etiology is often multifactorial, and its clinical presentation can range from asymptomatic to severe neurological symptoms, or even death (Hall & Guyton, 2016; Mount, 2018; Verbalis, 2003). The most common cause of hyponatremia is osmotically inappropriate (hereafter: inappropriate) release of arginine vasopressin (AVP) by the posterior pituitary gland, which results in excessive renal water reabsorption by stimulating translocation of aquaporins (Hall & Guyton, 2016; Mount, 2018). This non‐osmotic AVP release can be caused by intravascular hypovolemia, and—more commonly—the syndrome of inappropriate antidiuresis (SIAD), which has an extensive differential diagnosis (Hyponatremia, n.d.; Clarkson et al., 2010; Hall & Guyton, 2016; Mount, 2018). The net effect of a rise in AVP release on the collecting ducts is accurately reflected by an increase in urine osmolality ($O_{u}$), which determines the solute‐free water (SFW) balance that also takes into account urea and other osmotically inert solutes, as opposed to the EFW balance that only includes urine electrolytes (Hall & Guyton, 2016; Mount, 2018; Verbalis, 2003; Voets & Vogtländer, 2019).

If hyponatremia is present, the physiological response with regard to the osmoregulation is the efficient excretion of superfluous water by the kidneys, which ideally results in maximum urine dilution (a principle which is employed by the “water‐loading test” to differentiate SIAD from other causes of hyponatremia) (Hall & Guyton, 2016; Mount, 2018; Pliquett & Obermuller, 2017). Physiological or “osmotic” AVP secretion is negligible at plasma osmolality values below the hypothalamic osmotic threshold of approximately 280 mOsmol/kg (Hall & Guyton, 2016; Mount, 2018; Voets et al., 2021). Thus, inappropriate AVP release is considered to be present when $O_{u}>O_{u,\min}$ or $O_{u}>100$ mOsmol/kg in the context of hyponatremia, since urine leaving Henle's loop and before entering the collecting duct is diluted to its minimum osmolality of approximately 100 mOsmol/kg (although some inter‐individual variation exists) (Hall & Guyton, 2016; Mount, 2018; Voets et al., 2021). Prevailing clinical hyponatremia guidelines, protocols and flowcharts employ this principle by postulating that hyponatremia with a $O_{u}$ value >100 mOsmol/kg or a $O_{u}$ value higher than the plasma osmolality ($O_{p}$) should suggest a monofactorial diagnosis of inappropriate AVP release (Ter Maaten et al., 2017–2021; Hoorn et al., 2014; Hoorn & Zietse, 2017; Sterns et al., 2022; Workeneh et al., 2023). Unfortunately, the clinical practice tends to be more complex, and this over‐simplification comes at the cost of loss of nuance. The monofactorial “quick‐and‐dirty approach” in many of these widely consulted clinical resources, although convenient, has important caveats from a physiological viewpoint. As mentioned before, the etiology of hyponatremia is often multifactorial, and only a minority is attributable to a single cause. Buchkremer et al. (2023) recently established the cause or causes of hyponatremia in 279 patients, based on the general principle of EFW balance as presented in Equation (1) in the next section. They demonstrated that for SIAD patients, negative EFW clearance due to inappropriate antidiuresis is the sole cause of hyponatremia in only 30.7% of patients, whereas high EFW intake is an additional factor in almost 70% of cases (Buchkremer et al., 2023). For hypovolemic and diuretic‐induced hyponatremia, inappropriate antidiuresis alone was found to be the cause in only 14.8% and 22.5%, respectively (Buchkremer et al., 2023). Thus, while analyzing hyponatremia, it is imperative to not only verify that a certain degree of inappropriate AVP release is present (“concurrent AVP release”), but also to ascertain whether it *in itself*—that is, in the absence of significant hypotonic fluid intake—can sufficiently explain the observed hyponatremia (“sufficient AVP release”) in order to understand the pathophysiological mechanism(s) at play. As will be argued in this report, these two are not necessarily synonymous (even though any degree of inappropriate AVP release naturally contributes to the occurrence and/or the perpetuation of hyponatremia to some extent, since renal water clearance would be more efficient if it were absent). For instance, the majority of clinicians would intuitively agree that a SIAD with a $O_{u}$ value of 750 mOsmol/kg is a satisfactory monofactorial explanation for an observed hyponatremia of 120 mmol/L, whereas a $O_{u}$ value of 300 mOsmol/kg seems unlikely as a sole etiology, even though the aforementioned guideline criteria of $O_{u}>100$ mOsmol/kg, and $O_{u}>O_{p}$ are met in both examples (Ter Maaten et al., 2017–2021; Hoorn et al., 2014; Hoorn & Zietse, 2017; Sterns et al., 2022; Workeneh et al., 2023). In the latter case, many clinicians would be instinctively reluctant to accept inappropriate AVP release as the sole etiology, and would assume that the patient must also ingest a significant degree of hypotonic fluids. In the former example, the majority would probably not consider additional hypotonic fluid intake a requirement: the sheer degree of renal water retention in itself would be deemed a sufficient explanation for the observed hyponatremia. This begs the question: can a line be rationally drawn between these scenarios in order to substantiate this clinical intuition?

The major problem with the abovementioned premise in many clinical guidelines is that while the degree of AVP release is best reflected by $O_{u}$, which is an marker of the SFW balance, the plasma sodium concentration is reflected much more accurately by the EFW balance (Hall & Guyton, 2016; Shah & Bhave, 2018; Shimizu & Kurosawa, 2002; Voets & Vogtländer, 2019). The novel criterion proposed in this report utilizes some of the same concepts from renal physiology as the well‐known Furst model for rational fluid restriction, but with a different objective: it aims to distinguish absent, concurrent and sufficient AVP release in a hyponatremic patient by using the concept of theoretical maximum urine tonicity (which will be clarified hereafter) to bridge the gap between the EFW balance as a predictor of the plasma sodium course, and $O_{u}$ as a measure of inappropriate AVP release (Furst et al., 2000; Musch & Decaux, 1998; Voets & Vogtländer, 2019). Rather than calculating a specific EFW balance at a specific point in time, this criterion sets the ceilings for three different hyponatremia scenarios based on the same, easily obtainable plasma and urine parameters, and strives to further establish the etiology of hyponatremia.

A stepwise mathematical derivation is presented below.

## 2 CRITERION DERIVATION

A *condictio* *sine qua non* for the occurrence and/or exacerbation of hyponatremia is an increase in EFW, so: ∆EFW>0 (Hall & Guyton, 2016; Mount, 2018; Shimizu & Kurosawa, 2002; Verbalis, 2003; Voets & Vogtländer, 2019). An exception to this rule is translocational hyponatremia, often as the result of hyperglycemia (although this type of hyponatremia is generally non‐hypotonic) (Hall & Guyton, 2016; Mount, 2018). This change in EFW (∆EFW) equals the difference between EFW intake (EFWI) and EFW clearance (EFWC): (Shah & Bhave, 2018; Shimizu & Kurosawa, 2002; Voets & Vogtländer, 2019)

(1) ∆EFW=EFWI−EFWC

Insensible water losses, such as through perspiration and/or respiration, are disregarded here for the sake of this model. According to the well‐known definitions, EFWI and EFWC can be expressed mathematically as follows: (Furst et al., 2000)

(2) $$\text{EFWI}=V_{\text{in}}\left(1-\frac{{\left[{\mathrm{Na}}^{+}+K^{+}\right]}_{\text{in}}}{{\left[{\mathrm{Na}}^{+}\right]}_{p}}\right)$$

(3) $$\text{EFWC}=V_{u}\left(1-\frac{{\left[{\mathrm{Na}}^{+}+K^{+}\right]}_{u}}{{\left[{\mathrm{Na}}^{+}\right]}_{p}}\right)$$

**FIGURE 1:**
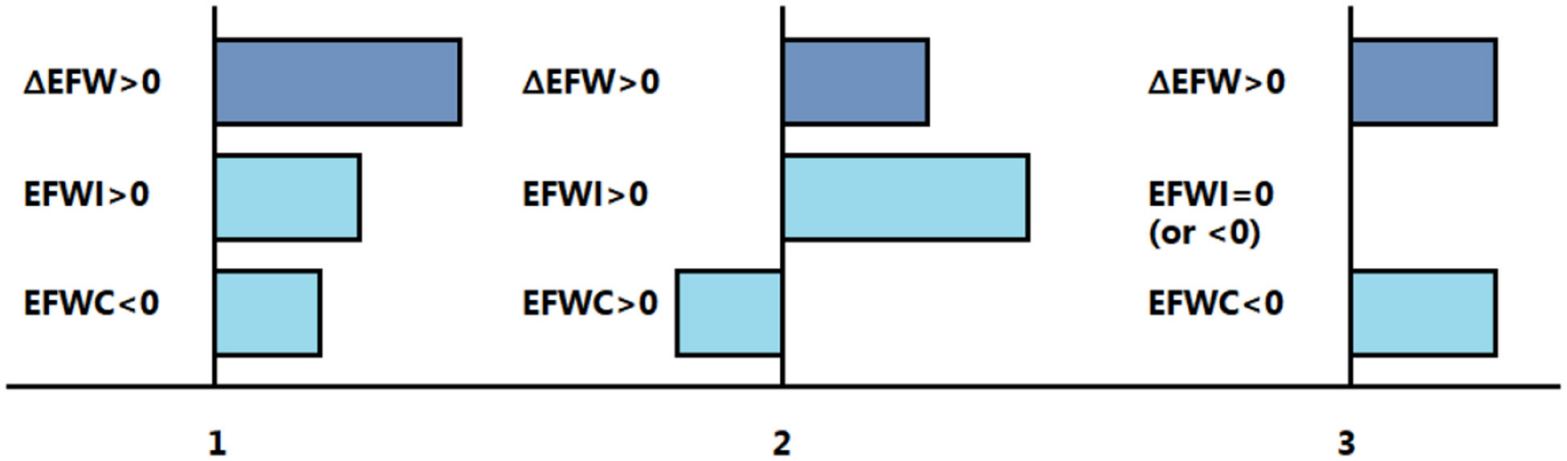
Schematic representation of the three scenarios described in the text in which hypotonic hyponatremia ensues because ∆EFW>0. The bars on the left side of the midline represent electrolyte‐free water (EFW) loss, whereas the bars on the right side of the midline represent EFW gain. Scenario 1 (left) describes a combined positive EFW intake (EFWI>0) and a negative EFW clearance (EFWC<0); for example, multifactorial etiology. Scenario 2 (center) describes a positive EFW intake (EFWI>0), which exceeds a positive EFW clearance (EFWC>0); for example, primary polydipsia. Scenario 3 (right) describes a negative EFW clearance (EFWC<0), and a neutral (or smaller negative) EFW intake (EFWI≤0), for example, SIAD. EFWI<0, although unlikely in the context of pre‐treatment hyponatremia, holds true for relatively hypertonic intake.

Although several variations on the equations above have been proposed over the years, such as the more convoluted “modified EFWC” formula or MEFWC formula, there is little evidence to support their use over the more elegant equations above (Lindner & Schwarz, 2012; Nguyen & Kurtz, 2005). Based on Equation (1), three separate scenarios (1–3) for ∆EFW>0 can be distinguished (Figure 1):

1. EFWI>0,and EFWC<0
2. EFWI>0,EFWC≥0,and EFWI>EFWC
3. $\text{EFWC}<0,\text{EFWI}\leq 0,\text{and}\;\left|\text{EFWC}\right|>\left|\text{EFWI}\right|$

As mentioned earlier, not every degree of inappropriate AVP release can be considered a solely sufficient explanation for hyponatremia. There are myriad hyponatremia cases where urine is concentrated to $O_{u}>100$ mOsmol/kg by relatively low‐grade, inappropriate AVP release, while EFWC≥0 is still true (Hall & Guyton, 2016; Mount, 2018; Voets & Vogtländer, 2019). In such a case, hyponatremia can only be explained by the second scenario mentioned above, and EFWI>0 (e.g., significant (sub)conscious polydipsia or infusion of hypotonic fluids) also needs to hold true in order to achieve ∆EFW>0, and thus a decrease in the plasma sodium concentration (Ter Maaten et al., 2017–2021; Clarkson et al., 2010; Hall & Guyton, 2016; Mount, 2018). Here, the AVP release is “concurrent” rather than “sufficient”, as it cannot in itself cause hyponatremia, which necessarily leads to the diagnosis of multifactorial hyponatremia. It is therefore incorrect to assume that inappropriate AVP release is by definition the sole etiology of hyponatremia, simply because urine is concentrated to a value above 100 mOsmol/kg, as is suggested in several diagnostic hyponatremia guidelines, or even to a value above the plasma osmolality, as is suggested in several others (Ter Maaten et al., 2017–2021; Hoorn et al., 2014; Hoorn & Zietse, 2017; Sterns et al., 2022; Workeneh et al., 2023). Inappropriate AVP release in itself can only be a sufficient cause of hyponatremia if the conditions of the third scenario above are met (assuming neutral intake (EFWI=0), since relevant hypertonic intake (EFWI<0) would not be reasonably expected during the development of hyponatremia), namely: Shah & Bhave, 2018.

(4) $$\begin{array}{c} \text{EFWC}=V_{u}\left(1-\frac{{\left[{\mathrm{Na}}^{+}+K^{+}\right]}_{u}}{{\left[{\mathrm{Na}}^{+}\right]}_{p}}\right)=V_{u}\left(1-\frac{{\left[E^{+}\right]}_{u}}{{\left[{\mathrm{Na}}^{+}\right]}_{p}}\right)\\ =V_{u}\left(1-\frac{2{\left[E^{+}\right]}_{u}}{2{\left[{\mathrm{Na}}^{+}\right]}_{p}}\right)=V_{u}\left(1-\frac{T_{u}}{T_{p}}\right)<0 \end{array}$$

In the equation above, the factor 2 accounts for anions in both urine and plasma, and $T_{u}$ and $T_{p}$ correspond to the urine tonicity and plasma tonicity (which equals approximately twice the plasma sodium concentration, unless hyperglycemia is present), respectively (Voets & Vogtländer, 2019). Urine osmolality is the sum of osmotically active solutes, referred to as urine tonicity, and osmotically inert solutes, such as urea. Since urine always contains a certain amount of osmotically inactive solutes, the value for urine tonicity will necessarily be lower than the value for urine osmolality. For the theoretical maximum urine tonicity ($T_{u,\max}$) for a given $O_{u}$ value this means: (Musch & Decaux, 1998; Shimizu & Kurosawa, 2002; Voets & Vogtländer, 2019).

(5) $$T_{u,\max}=2\cdot {\left[{\mathrm{Na}}^{+}+K^{+}\right]}_{u,\max}=C\cdot O_{u}\;\text{with}\;0<C<1$$

**FIGURE 2:**
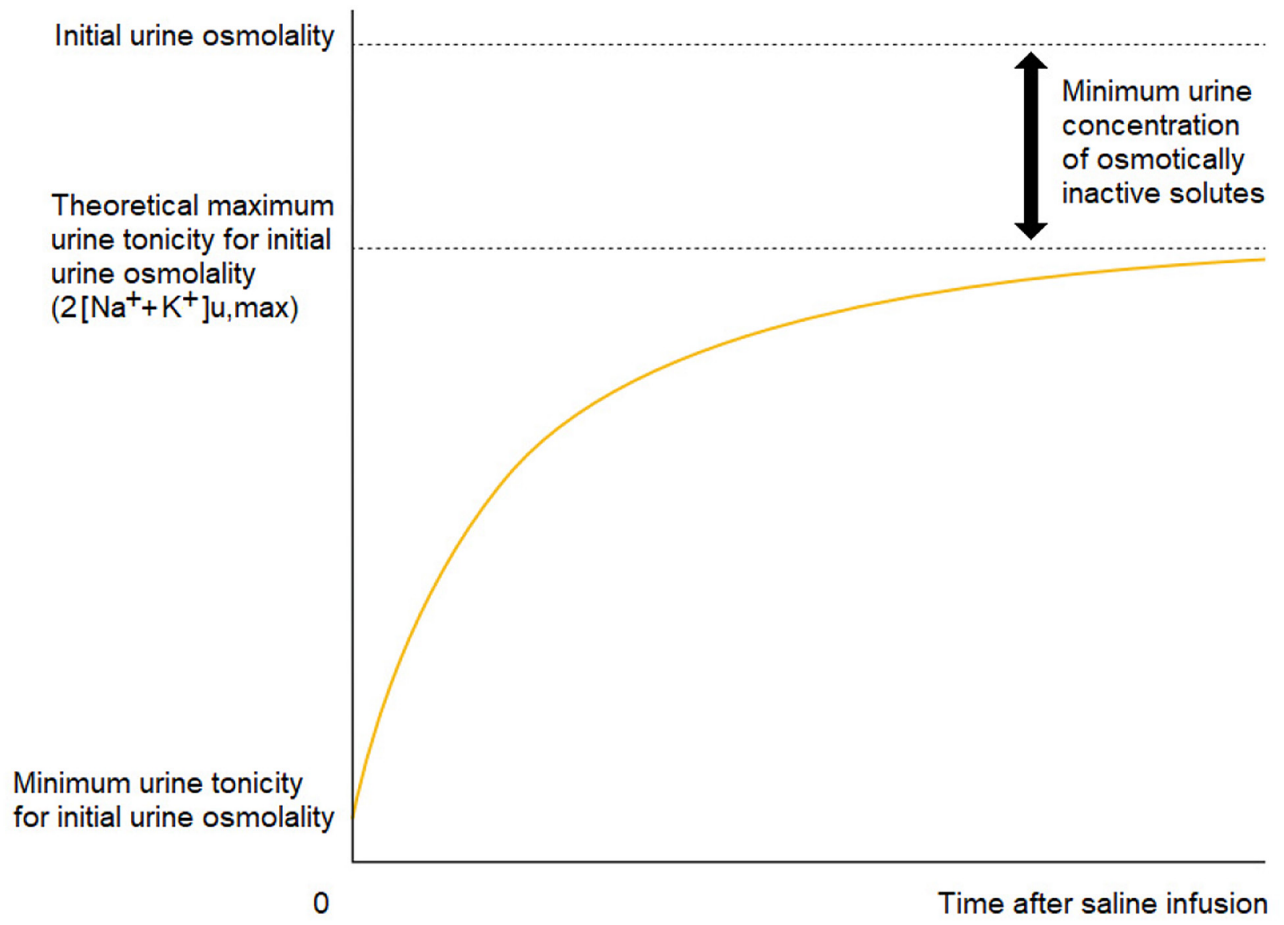
Graphical representation of the concept of theoretical maximum urine tonicity ($T_{u,\max}=2{\left[Na^{+}+K^{+}\right]}_{u,\max}$), as first introduced by Musch and Decaux (1998) (Shimizu & Kurosawa, 2002; Voets & Vogtländer, 2019). This graph shows a simplified model of how the urine electrolyte concentration (representing the percentage urine tonicity for a given urine osmolality, that is, $2{\left[Na^{+}+K^{+}\right]}_{u}$) can fluctuate in time, depending on intake and/or infusion. Because at least a minimum amount of non‐electrolyte solutes (such as urea) needs to be excreted in urine, $T_{u,\max}$ is necessarily lower than $O_{u}$. Therefore, the urine electrolyte concentration must always remain between a lower limit and an upper limit within a given urine osmolality value, as expressed in Equation (5) in the main text. $T_{u,\max}$, which is achieved after prolonged saline infusion, can be estimated from the initial urine osmolality (approximately 60% of $O_{u}$), and has been found to correlate much more strongly with the change in plasma sodium concentration than random ${\left[Na^{+}+K^{+}\right]}_{u}$ values (Musch & Decaux, 1998; Shimizu & Kurosawa, 2002; Voets & Vogtländer, 2019).

The lowest value for EFWC (and thus the largest renal water reabsorption) for a certain urine osmolality is achieved when the percentage of osmotically active solutes in urine is at its maximum, which means that $T_{u}=T_{u,\max}$. Both Shimizu et al. and Musch et al. have investigated the value for $T_{u,\max}$ for a give urine osmolality by measuring the urine composition after several hours of saline infusion, and established that—for any given urine osmolality—$T_{u,\max}$ constitutes approximately 60% of the initial urine osmolality under normal dietary conditions; they concluded that $2\cdot {\left[{\mathrm{Na}}^{+}+K^{+}\right]}_{u,\max}/O_{u}$ equals 59.7 ± 1.7%, and that ${\left[{\mathrm{Na}}^{+}+K^{+}\right]}_{u,\max}/O_{u}$ equals 33.0 ± 10.0%, respectively (Figure 2): (Musch & Decaux, 1998; Shimizu & Kurosawa, 2002; Voets & Vogtländer, 2019)

(6) $$T_{u,\max}=2\cdot {\left[{\mathrm{Na}}^{+}+K^{+}\right]}_{u,\max}\approx 0.6\cdot O_{u}$$

For the sake of this model, the abovementioned value will be used, so C=0.6, although it is important to note that every realistic value for C could be used without changing this criterion's core derivation (Musch & Decaux, 1998; Shimizu & Kurosawa, 2002; Voets & Vogtländer, 2019). EFWC, as defined in Equation (3), will become negative, and thus EFW will be retained by the kidneys (allowing hyponatremia to ensue), according to the third scenario above, if –and only if:

(7) $$T_{u,\max}=0.6\cdot O_{u}>T_{p}=2\cdot {\left[{\mathrm{Na}}^{+}\right]}_{p}$$

(8) $$O_{u}>\frac{2}{0.6}\cdot {\left[{\mathrm{Na}}^{+}\right]}_{p}=3.3\cdot {\left[{\mathrm{Na}}^{+}\right]}_{p}$$

If the condition EFWC<0 is not met at $T=T_{u,\max}$ for a certain value for $O_{u}$, then it will definitely not be met at values of $T<T_{u,\max}$ for the same $O_{u}$. Therefore, AVP release has to concentrate urine to at least $O_{u}>3.3\cdot {\left[{\mathrm{Na}}^{+}\right]}_{p}$ in order to produce and/or aggravate hyponatremia without the necessity for EFWI>0.

Based on the degree of AVP release, again three hyponatremia scenarios (A–C) can be distinguished, where the commonly used cut‐off value $O_{u}<100$ mOsmol/kg is taken as evidence of AVP absence. This trichotomy is as follows:

- $O_{u}<100$ *mOsmol/kg*: no (relevant) AVP release is present, thus EFWC>0 (an appropriate response), and hyponatremia must result from EFWI>EFWC (the previously described scenario 2), as is the case in e.g. primary polydipsia or tea‐and‐toast syndrome ➔ *Absent AVP release*
- $100<O_{u}<3.3\cdot {\left[{\mathrm{Na}}^{+}\right]}_{p}$ *mOsmol/kg*: *some* inappropriate AVP release is present, but since EFWC>0, it is definitely unable to produce hyponatremia *in itself* without the condition EFWI>0 being also met ➔ *Concurrent AVP release*
- $O_{u}>3.3\cdot {\left[{\mathrm{Na}}^{+}\right]}_{p}$ *mOsmol/kg*: inappropriate AVP release such that EFWC≤0 is present, theoretically *in itself* able to produce hyponatremia (barring major hypertonic intake, which would not be logically expected during the occurrence or aggravation of hyponatremia). However, other factors causing EFWI>0 cannot be ruled out (especially an $O_{u}$ value only slightly larger than the criterion's upper limit with severe hyponatremia suggests that other factors have contributed as well, or that this degree of inappropriate AVP release has existed for a long time) ➔ *Sufficient AVP release*

From the mathematical expression of EFWC, it intuitively follows that the plasma sodium concentration dictates when EFWC<0 is reached (Shah & Bhave, 2018; Voets & Vogtländer, 2019). Also, the more severe the hyponatremia, the more readily should any degree of AVP release be considered abnormal. By ignoring the relatively low plasma potassium concentration in this formula, the upper limit in scenario B is slightly lower than it would be if plasma potassium were incorporated, thus lowering the chance that AVP release is falsely considered “concurrent”, rather than “sufficient”.

## 3 DISCUSSION

In the previous section, a novel criterion has been proposed to differentiate absent, concurrent, and sufficient AVP release in a hyponatremic patient. This could be an addition to the current diagnostic hyponatremia flowcharts: not only is the *presence* of AVP release established, its *relevance* is examined as well, further clarifying the underlying pathophysiological mechanism(s) at play during the occurrence and/or exacerbation of hyponatremia. This criterion aims to serve as a reminder for the clinician to keep considering all relevant aspects of the EFW balance in the—often complex—analysis of hyponatremia (somewhat analogous to the delta ratio in the analysis of multifactorial metabolic acid–base disturbances) (Ter Maaten et al., 2017–2021). Its derivation rests on two main pillars, namely the observation that not every degree of urine concentration or AVP release is enough to explain a net EFW gain (contrary to what is often erroneously suggested in clinical guidelines and protocols), and using existing concepts from renal physiology to combine EFW balance and SFW balance and to set the physiological ceilings for the three aforementioned hyponatremia scenarios A–C.

If the measured $O_{u}$ value in a hyponatremic patient is below the lower limit in scenario A (e.g., 75 mOsmol/kg), then no AVP release is present, urine is appropriately dilute, and the intake of hypotonic fluids is simply too large for the kidneys to handle (either due to its sheer volume or due to the inability of the kidneys to produce an adequate volume of urine). If $O_{u}$ is between the lower and upper limit in scenario B, then it can be concluded that while a certain degree of AVP is present, it is merely contributory, rather than explanatory, for the observed hyponatremia. Other factors should be actively sought and treated: the AVP release *itself* is simply unable to produce hyponatremia, because the lowest achievable EFWC value for the given $O_{u}$ is still positive (and would definitely be positive at higher plasma sodium concentrations earlier in the course of hyponatremia). Recall the previously discussed hyponatremic patient with a plasma sodium concentration of 120 mmol/L, and a $O_{u}$ of 300 mOsmol/kg (Table 1). Since 100<300<3.3·120, it can be concluded that while some AVP release is present, it is insufficient as a sole cause for the hyponatremia, and EFWI>0 *must* also hold true and be treated. Lastly, if the measured $O_{u}$ value in a hyponatremic patient is above the criterion's upper limit in scenario C, the AVP release *could* solely explain the hyponatremia, barring significant hypertonic intake (which would not be expected during the occurrence of hyponatremia), and not excluding other possibly contributory factors. If the criterion is applied to the same patient, but this time with a $O_{u}$ of 750 mOsmol/kg, it can easily be seen that 750>3.3·120, and thus this degree of inappropriate antidiuresis could well be the sole cause, although this conclusion cannot be drawn as firmly as in the scenarios A and B.

**TABLE 1:** Schematic overview of the physiological interpretation of the plasma and urine parameters in the three hypothetical hyponatremia scenarios (as discussed in the main text), based on the prevailing clinical guideline recommendations, and on the proprosed criterion.

Clinical scenario	A	B	C
${\left[Na^{+}\right]}_{p}$	120 mmol/L	120 mmol/L	120 mmol/L
$O_{u}$	75 mOsmol/kg	300 mOsmol/kg	750 mOsmol/kg
$O_{u}>100$ mOsmol/kg	No	Yes	Yes
$O_{u}>O_{p}$	No	Yes	Yes
$O_{u}>3.3{\left[Na^{+}\right]}_{p}$	No	No	Yes
Physiological interpretation (criterion)	Absent AVP release	Concurrent AVP release	Sufficient AVP release

Abbreviations: AVP, arginine vasopressin; ${\left[Na^{+}\right]}_{p}$, plasma sodium concentration; $O_{u}$, urine osmolality; $O_{p}$, plasma osmolality.

It should be noted that the urine composition tends to fluctuate over time, depending on food intake, saline infusion, aldosterone release, and many other factors. In the case of non‐osmotic, and relatively feedback‐independent AVP release, the value for $O_{u}$—while not as “fixed” as previously thought—has the advantage of being more stable over time than the specific urine electrolyte components, but is inferior to the EFW balance as a predictor of the plasma sodium course (Clarkson et al., 2010; Hall & Guyton, 2016; Verbalis, 2003; Voets & Vogtländer, 2019). The parameter $T_{u,\max}$, as estimated from $O_{u}$, combines these advantages, but does remain subject to a degree of fluctuation in time, albeit to a lesser extent than the urine electrolyte concentrations (such as in the Furst ratio) (Clarkson et al., 2010; Hall & Guyton, 2016; Verbalis, 2003; Voets & Vogtländer, 2019). Fortunately, the presented criterion aims to further elucidate the underlying etiology of hyponatremia rather than quantify the EFW balance at a specific point in time. A model incorporating EFW intake would be practically unfeasible, because its exact electrolyte composition is often unknown.

In conclusion, the derived criterion aims to determine when a suggested diagnosis of “monofactorial hyponatremia” should be taken with a grain of salt. It can hopefully facilitate the interpretation of plasma and urine parameters in a patient presenting with hyponatremia, and warn against the important clinical pitfall of attributing hyponatremia too readily to a single cause.

## 4 AUTHOR CONTRIBUTIONS

PJGMV conducted the basic research, drafted and revised the main text, and created and revised the figures and table.

## FUNDING INFORMATION

The author declares to have received no financial support.

## CONFLICT OF INTEREST STATEMENT

The author has declared that no conflict of interest exists.
